# Viruses in the Built Environment (VIBE) meeting report

**DOI:** 10.1186/s40168-019-0777-4

**Published:** 2020-01-04

**Authors:** Aaron J. Prussin, Jessica A. Belser, Werner Bischoff, Scott T. Kelley, Kaisen Lin, William G. Lindsley, Jean Pierre Nshimyimana, Michael Schuit, Zhenyu Wu, Kyle Bibby, Linsey C. Marr

**Affiliations:** 1 Department of Civil and Environmental Engineering, Virginia Tech, Blacksburg, VA 24061 USA; 2 Influenza Division, National Center for Immunization and Respiratory Diseases, Centers for Disease Control and Prevention, Atlanta, GA 30333 USA; 3 Section on Infectious Diseases, Wake Forest School of Medicine, Winston-Salem, NC 27157 USA; 4 Department of Biology, San Diego State University, San Diego, CA 92182 USA; 5 Health Effects Laboratory Division (HELD), National Institute for Occupational Safety and Health, Morgantown, WV 26505 USA; 6 Fisheries and Wildlife, Michigan State University, East Lansing, MI 48823 USA; 7 National Biodefense Analysis and Countermeasures Center, Frederick, MD 21702 USA; 8 Department of Civil and Environmental Engineering and Earth Sciences, University of Notre Dame, Notre Dame, IN 46556 USA

**Keywords:** Virus, Virome, Built environment, Exposure, Metagenomics, Disease transmission

## Abstract

**Background:**

During a period of rapid growth in our understanding of the microbiology of the built environment in recent years, the majority of research has focused on bacteria and fungi. Viruses, while probably as numerous, have received less attention. In response, the Alfred P. Sloan Foundation supported a workshop entitled “Viruses in the Built Environment (VIBE),” at which experts in environmental engineering, environmental microbiology, epidemiology, infection prevention, fluid dynamics, occupational health, metagenomics, and virology convened to synthesize recent advances and identify key research questions and knowledge gaps regarding viruses in the built environment.

**Results:**

Four primary research areas and funding priorities were identified. First, a better understanding of viral communities in the built environment is needed, specifically which viruses are present and their sources, spatial and temporal dynamics, and interactions with bacteria. Second, more information is needed about viruses and health, including viral transmission in the built environment, the relationship between virus detection and exposure, and the definition of a healthy virome. The third research priority is to identify and evaluate interventions for controlling viruses and the virome in the built environment. This encompasses interactions among viruses, buildings, and occupants. Finally, to overcome the challenge of working with viruses, workshop participants emphasized that improved sampling methods, laboratory techniques, and bioinformatics approaches are needed to advance understanding of viruses in the built environment.

**Conclusions:**

We hope that identifying these key questions and knowledge gaps will engage other investigators and funding agencies to spur future research on the highly interdisciplinary topic of viruses in the built environment. There are numerous opportunities to advance knowledge, as many topics remain underexplored compared to our understanding of bacteria and fungi.

Video abstract.

## Introduction

Research on the microbiology of the built environment has grown swiftly in recent years, catalyzed by advances in sequencing and metagenomic analyses and investment from the Alfred P. Sloan Foundation to nurture a new multidisciplinary field of scientific inquiry. Although microbiology encompasses the study of bacteria, fungi, and viruses, to date, most studies involving the built environment have focused on bacteria and fungi while largely overlooking viruses, which have been described as “the forgotten siblings of the microbiome family” [1]. Viruses are as numerous as bacteria in indoor air [2], and viruses merit attention because of their importance to human health [3] and role in overall microbial ecology [4–6].

Efforts to study the viral community, or virome, can build upon the research agenda presented in the National Academies of Sciences, Engineering, and Medicine’s report on microbiomes of the built environment [7]. The report identified 12 priority areas, of which several are especially pertinent to viruses. For example, understanding the interrelationships among microbial communities, human occupants, and buildings should include viruses as well as bacteria and fungi. Due to the inherent technical difficulty in studying viruses [8], advances are needed in methods and tools to detect and identify them.

## Meeting format

The Viruses in the Built Environment (VIBE) meeting was sponsored by the Alfred P. Sloan Foundation and took place during May 2019 in Arlington, Virginia. Twenty-seven researchers from the USA studying different aspects of viruses in the built environment were invited to participate. Their expertise spanned environmental engineering, environmental microbiology, epidemiology, infection prevention, fluid dynamics, occupational health, metagenomics, and virology. Representatives from academia, government, and funding agencies participated in the meeting.

Presentations and discussions during the meeting were organized around three themes: (1) sources, transformation, and transport of viruses in the built environment; (2) viral metagenomics; and (3) transmission and ecology. The first session highlighted the advantages and disadvantages of current airborne virus sampling methods, the impact of virus structure on fate in the environment, and the spread of respiratory droplets indoors. The second session addressed the potentials and pitfalls of viral bioinformatics, metagenomic analysis of airborne viruses in a dormitory, and the potential of utilizing crAssphage as an indicator to study the virome in the built environment. The third session summarized the application of aerobiological techniques to improve studies of influenza transmission in the ferret model, the role of droplet composition in respiratory disease transmission, and virus detection in the healthcare environment. Finally, participants identified key research questions for studying viruses in the built environment.

The specific aims of this workshop were to (1) generate an interdisciplinary review of the current state of knowledge on viruses in the built environment, (2) identify key research questions and funding priorities, and (3) raise awareness about the need for research on viruses in the built environment.

## Summary of key research questions and funding priorities

### Viral community in the built environment

#### Basic questions about viral communities in the built environment

Compared to our knowledge about bacterial and fungal communities in the built environment [9], we know very little about viral communities. Metagenomic approaches now allow for identification of numerous viruses at once, but researchers are still limited by reference databases. In addition, metagenomic identification typically does not definitively identify viral hosts. As these databases expand, we will be better able to answer the “who’s there?” question about viruses.

While cataloguing the diversity of viruses in different types of built environments is of fundamental interest, more targeted questions regarding viral activity and transport in the built environment are likely of more immediate applicability. Still, questions remain about how many virus species we have not yet identified and how our knowledge is biased by sampling and analytical methods. Beyond qualitative information, we would like to know the concentrations of specific viruses in the built environment, in air and on different types of surfaces, and whether the total is dominated by bacteriophages or human, animal, or plant viruses. Furthermore, it is possible that only a portion of the viral community may be infectious, while the remainder is “inert.” Combining viral and bacterial community information with knowledge about the microorganisms’ activity will help us determine the role of viruses in the built environment.

Viruses typically are tens to hundreds of nanometers in size and are usually associated with environmental debris. Aerosol-generating processes such as coughing, toilet flushing, and dust resuspension can generate a broad size range of virus-laden airborne particles that also include salts, mucus, proteins, cellular debris [10, 11], and other components. Consequently, most airborne viruses are usually associated with particles that are much larger than the viruses themselves. For example, the influenza virus is about 0.1 μm in diameter, but studies of various indoor environments have found that the majority of airborne virus is associated with particles larger than 1 μm in diameter [12, 13]. Knowing the size of virus-laden particles is critical for predicting their transport and fate.

#### Viral community dynamics

Very little is known about viral community dynamics and how communities vary in both time and space. Studies have shown that the bacterial and fungal microbial communities are geographically patterned in the built environment [14]; such investigations have not been conducted for viruses. Understanding the seasonality of the virome in the built environment is of keen interest, since it might help explain patterns of illness that are observed throughout the year (e.g., influenza outbreaks during winter). A recent study of the airborne virome in a daycare center found that viral communities varied by season [15], in contrast to bacterial communities in air and dust, which do not appear to shift by season [15, 16]. We have yet to identify the major driver of the virome in the built environment. It is likely to be a combination of geography, timing, architectural design, and occupants’ activities. By deciphering the effect of each component on the virome, we will improve our ability to predict the spatial and temporal dynamics of the viral community in the built environment.

#### Sources shaping the virus community

With the rapid explosion of metagenomic approaches, we are beginning to understand the sources of viruses in the built environment. These may include humans; pets; plants; plumbing systems; heating, ventilation, and air-conditioning (HVAC) systems; mold; dust resuspension; and the outdoor environment [17]. A study using shotgun metagenomics [18] found that viruses in a college dormitory originated from many different organisms, including animals, arthropods, bacteria, fungi, humans, plants, and protists. Considering the constant movement of people and air between indoors and outdoors, we can assume that the outdoor environment influences the viral community in the built environment. A recent study examining the seasonality of viruses in a daycare center found that outdoor/plant-associated viruses played a large role in shaping the viral community in spring and summer, when windows and doors were open more frequently [15]. A better understanding of how different sources shape the viral community could enable interventions to select for a desirable microbiome, ultimately leading to healthier buildings.

#### Virus-bacteria community interactions

While the bacterial and fungal communities in the built environment have been studied extensively, knowledge of their interactions with viral communities is lacking, mainly due to the hurdles in viral sequencing toolkits. However, mounting evidence indicates that the interconnectivity between the viral community and other microbial communities (i.e., virus-virus, bacteria-virus interactions, and fungi-virus interactions) is an important driver of the microbial evolutionary process [19] and has significant implications for human health [20]. Recent studies have not only demonstrated phage therapy as an effective approach in combating bacterial infection [21, 22] but have also revealed that bacteria-virus and virus-virus interactions can affect the pathogenesis of diseases [23–25]. Researchers need to examine the interactions among bacteria, fungi, and viruses in the built environment, preferably at the community level, and the evolution of the microbiome as the structure of each component dynamically shifts.

### Health

#### Healthy virome

Historically, viruses have been viewed as threatening because they were best known for causing disease. While their full role in human health is still mostly unknown [26, 27], we are beginning to understand the associations between the enteric and respiratory virome and acute and chronic human diseases [27–30], and a recent study showed that bacteriophages modulate bacteria communities in the gut [31]. The majority of viruses and virus-derived genetic elements appear to be benign; some may even be essential for good health if the hygiene hypothesis [32] applies to viruses as well as bacteria. This leads to a critical question: is there a healthy virome, and if so what is it? Researchers have discovered many beneficial viruses and have identified mutualistic relationships between viruses and a wide range of hosts [33]. A recent study has shown that healthy individuals across the globe share a core and common set of bacteriophages in the gut [34], evidence supporting the concept of a healthy human gut virome. As information about potentially beneficial viruses becomes more available, researchers should focus on defining a healthy virome of the built environment and determining whether we can manipulate the viral community, as has been shown for the bacterial community [35, 36].

#### Role of bacteriophages

The role that bacteriophages play in microbial ecology in the built environment is also unknown. Viruses are numerous in the built environment: in indoor air; the concentrations of virus-like particles and bacteria-like particles are comparable [2]. Overall microbial activity is low in buildings without water damage [37], suggesting that bacteriophages in buildings are likely dormant. It is possible that phage therapy, the use of bacteriophages to treat bacterial infections in humans, could be extended to manipulate the bacterial community in the built environment. This would be especially desirable in a healthcare setting for the control of multidrug-resistant bacteria.

#### Relationship between virus detection and exposure risk

Following the classic disease-centered approach, researchers have traditionally focused on viruses that cause a specific illness. This focus has driven the development of treatments such as antivirals and preventive measures including gloves, gowns, and masks. Our growing appreciation of the importance of the human microbiome poses the challenge of determining if exposures to identified or yet unknown viruses should be promoted or hindered or will require a preventive or therapeutic response.

Estimating the risk of infection from viral pathogens requires knowledge of the association between the human infectious dosage (HID) and the transmission dynamics of a particular virus. Evidence of these interactions, however, is limited. For example, trials have provided some data on HID for respiratory viruses such as influenza, respiratory syncytial virus (RSV), and rhinoviruses, and for gastrointestinal viruses such as norovirus and rotavirus [38–42], but we do not know how these HIDs might vary by virus strain, exposure route, or the recipient’s condition, such as immune status or co-infections. Environmental factors including air and surface temperature, humidity, UV light exposure, and air speed also influence the infectivity of viruses [43–50]. The comparison of the environmental presence of a virus with its known HID may provide us with estimates, although indirect, of infection risks. To estimate inhalation dose, we can multiply the airborne concentration of a virus by deposition efficiency and respiratory minute volume, but assessing the risk of indirect contact exposure requires improved understanding of how humans interact with surface materials in the built environment and how viruses transfer between the skin and the materials [51, 52]. Several studies have documented the presence and amount of viruses in healthcare settings, mostly in the air [12, 13, 53–61]. For example, influenza has been detected and quantified in emergency rooms, inpatient wards, and waiting rooms [12, 13, 38, 53–57]. These data can be used to inform estimates of the risk for healthcare workers exposed during care activities and studies of the efficacy of interventions such as masks or air purification [62, 63]. Improved knowledge of the human virome and the relative contribution of transmission routes for different pathogens will better elucidate the public health risk posed by viruses in the environment.

#### Virus infectivity in the built environment

Not all pathogenic viruses detected in the built environment by molecular methods are infectious. Properties of the virus (including the presence or absence of a lipid envelope, viral stability in the environment, and infectious dose), host (including age and level of immunosuppression), environmental conditions (including temperature, relative humidity, and source of light), and the mode of transmission (including airborne, fomite, and water routes) all contribute to the capacity of a virus to maintain infectivity following release from an infected individual for sufficient duration to cause infection in a susceptible individual [64]. Further studies are needed to better understand how the diverse surface environments and fomites present in the built environment affect stability and/or inactivation of different viruses [65, 66]. These points about pathogenic viruses also apply more generally to viruses and their hosts (e.g., bacteriophages and their bacterial hosts).

#### Transmission of viruses

The most common source of viruses that infect people is other people. For example, people who are infected with respiratory viruses such as measles or influenza can produce droplets containing the virus when they cough or even just exhale [67–70]. These viruses can spread to other people by landing directly on them, settling onto surfaces that are then touched by hands, and floating through the air and being inhaled. People with gastrointestinal viruses such as norovirus [71] can deposit viruses onto fomites such as food, phones, tables, and doorknobs via unclean hands or vomiting, and others can then become infected by hand-to-mouth transfer of the viruses. Some research suggests that noroviruses also may spread by droplets produced during vomiting and the flushing of toilets; these droplets can then settle onto nearby surfaces or possibly be inhaled [72]. Most viruses are spread by multiple routes, and viral disease transmission can be difficult to trace. The relative importance of the different transmission pathways (especially transmission by inhalation of airborne droplets) often is unclear and sometimes is hotly debated [73].

### Interactions and interventions

#### Interactions among viruses, occupants, and buildings

There are complex and interdependent interactions among microbial communities, human occupants, and the built environment [7]. For example, human physiology, human-associated microorganisms, and human behavior affect the amount and types of microorganisms that are present in the built environment, ultimately shifting the viral community structure [74–76]. Abiotic factors, such as HVAC systems, plumbing and building materials, geographical location, and seasonality, can also affect the virome [15]. To date, studies have overlooked how the virome of the built environment differs between developed and developing countries, as well as how it varies by degree of urbanization, with varying architecture and building practices. Further, it would be interesting to understand how different cultural aspects (e.g., socioeconomic status, diet, occupation) affect the virome of the built environment. We are beginning to understand these complex interactions for bacteria [77], and fuller knowledge about such interactions for all types of microorganisms will enable us to improve the health of both humans and the built environment.

#### Built engineered systems

While recent studies have shed light on the microbiome of “traditional” built environments, including homes, offices, schools, medical facilities, and farms [55, 78] [79, 80], other types of built environments have received less attention. For example, very little is known about the virome of aquatic and outdoor built environments, such as aquatic engineered systems and water-based amusement parks created for recreation or food production. These types of systems can harbor viruses, as demonstrated in a study of aquatic built environments that linked aquarium operations to changes in viral ecology [78]. The United Nations Food and Agriculture Organization (FAO) has concluded that viral diseases are associated with global annual aquaculture losses of $6 billion [81–83]. Studies of these neglected engineered systems will provide knowledge to guide system engineering operations, promote disease prevention, and reduce economic losses.

#### Interventions

Several building management practices, including manipulation of ventilation rate, control of moisture, filtration of particles, use of UV germicidal irradiation, application of chemical disinfectants, and introduction of beneficial microorganisms, have been shown to be effective interventions to reduce microbial exposure risks and improve human health [7]. To date, studies have focused mainly on the effectiveness of interventions for removing biological particles that promote allergy symptoms and asthma development [84, 85]. It is not clear if these interventions might be effective for virus removal as well or whether modifications might be needed to generate a more desirable virome. A recent study showed that humidification of school classrooms was associated with a reduction in the number of influenza-like illnesses among students, suggesting that moisture control could be an effective approach to reduce the incidence of viral respiratory infections [86]. To better protect humans from viral infections in built environment, researchers should focus on rigorously examining the effectiveness of known interventions and proposing new interventions to control airborne and surface-borne viruses.

### Tools needed to enhance the study of viruses in the built environment

#### Sample preparation and bioinformatics

Viruses present unique challenges for bioinformatics analyses, particularly when attempting to develop a comprehensive profile of the virome in a given environment. There are many protocols for isolation and quantification of specific well-known viruses (e.g., norovirus) in built environments [87–89], but the deep-sequencing approaches of the type used to characterize whole microbial communities (bacteria, archaea, and fungi) are not as straightforward with viruses. Sampling of viruses in the built environment presents significant challenges due to their small size and low loading on surfaces and in the air [2, 87, 90, 91]. Some viruses have RNA, rather than DNA, as their genetic material, requiring the use of different sequencing library preparation approaches [8, 15, 90].

Another challenge of studying viromes in the built environment is that viruses lack a single conserved equivalent to the small subunit ribosomal RNA (16S/18S) gene used in microbial diversity studies [92]. Without any common conserved genes, PCR amplification using degenerate “universal” primers is not possible except within limited viral taxonomic groups. Thus, virome profiling necessitates the use of shotgun metagenomics techniques, in which libraries of random DNA fragments are generated from a sample and then sequenced on a next-generation sequencing platform. To identify the viruses in the sequenced sample, bioinformatic algorithms such as BLAST compare the fragments to existing viral databases and use the matches to identify the types of viruses present in the sample. With marker genes, it is possible to identify unknown/uncultured microorganisms and place them within a taxonomic group. However, in metagenomics, the results are almost entirely dependent on the quality and extent of the database, and if a fragment of DNA in a sequence does not have a match in a database, it is usually discarded. In many metagenomic studies, more than 50% of the sequences do not have a match and cannot be used for profiling [93]. This means that metagenomic virome profiling is largely dependent upon the accuracy and completeness of viral databases.

Viral genomes are also, on average, several orders of magnitude smaller than bacterial genomes [94]. This means that, given the same abundance of viral particles and bacterial cells in a community, the likelihood of sequencing a viral gene is 100 or 1000 times lower than for a bacterial gene. Many studies enrich the viral sequence fraction using size filtration to isolate viruses from bacteria and other cells, which also helps to ensure that the viral sequences come from free-living viruses rather than viral sequences integrated into bacteria or other host cells [92, 95]. However, extremely low viral (and total microbial) biomass in built environment surface and air samples makes filtration methods impractical.

The software algorithms used to perform viral database matching also deserve serious consideration, particularly with short-read sequencing data. Short sequences (100–200 nucleotides) provide limited information for pairwise alignments or for k-mer generation. While many researchers use automated workflows such as MG-RAST to analyze datasets, it is important to know how the algorithms work, the default settings, and the size and age of the databases used for matching. For instance, default BLAST e-values for a positive match with MG-RAST are very high (10^−5^), and likely to result in a lot of false positives [96]. For example, a recent analysis with MG-RAST in a mouse gut ecosystem identified a significant number of archaea in the samples [97]. However, a closer look at the data showed that, while the top hit to the supposed archaeal sequences was an archaeon, the next best match was often a bacterium. As with all bioinformatics or statistical methods, it is vital to understand the assumptions behind searches and know the default parameters of the methods. It is also highly recommended to double-check at least some results visually, particularly sequence alignments.

As databases, algorithms, and sequencing technologies improve, we expect viral metagenomics to become increasingly more useful and accurate. Viral genomes are being sequenced rapidly, and new approaches are starting to directly link viral genomes to host cells without the need for culturing [98]. Metagenome assembly methods continue to improve, allowing the generation of longer contiguous sequences (contigs) and even complete viral genomes directly from a sequencing dataset. These longer sequences not only greatly improve the confidence of matches but can also lead to the discovery of novel viruses [99].

#### Unculturable viruses

Detection and quantification of viral genomes or antigens in the environment is an important step in understanding the virome of a built environment, but it is not simply the presence and/or relative abundance of viruses that is of consequence. The activity of viruses depends on their infectivity, or ability to infect a host, whether that host is a human, plant, bacteria, or even another virus. Infectivity is typically measured in culture-based assays where susceptible host cells are infected and titers of infectious virus quantified by the effect on the cells as measured by plaques, cytopathic effect, or fluorescent foci. However, the infectivity of a virus in a well-defined laboratory assay setting may not correlate to dynamic real-world settings with fluctuating environmental conditions, chemical microenvironments, and host sensitivities. Furthermore, the appropriate host of the virus may not be known, and some viruses have proven to be unculturable or difficult to culture even in cases where the host is known [100–102]. As a result of these challenges, several culture-independent methods for evaluating viral infectivity have been proposed, typically using a measure of the integrity of one or more parts of the virus as a proxy for the infectivity of the virus as a whole [103–105]. For example, viability-PCR (v-PCR), using propidium monoazide (PMA) or other reagents, measures the relative abundance of viral particles with an intact capsid and/or envelope [106]. However, while this method may provide information about the state of the capsid/envelope and the portion of the genome matching the primers, it does not account for the possibility of defective interfering virus particles, and it is blind to the state of surface ligands, which may be necessary for successful infection. Viruses may be inactivated or rendered incompetent for infection through damage to one or more critical components, including genomic damage via UV light or harsh chemicals, disruption of the integrity of the capsid and/or envelope, or impairment of the ability of surface ligands to interact with cellular receptors resulting from enzymatic or chemical processes. Development of a culture-independent method that can simultaneously account for the integrity of all viral components necessary for infection would be a major advance for the study of viruses in the built environment.

#### Pathogenic viruses

In some studies, viruses that are pathogenic to humans may be of interest from the outset or be found during the course of a field survey. Appropriate precautions should be taken with any such viruses, particularly when there is a priori awareness that they may be present (e.g., in healthcare settings). It should be noted that work with some pathogenic viruses, including certain influenza viruses and hemorrhagic fever viruses, is restricted to specialized biocontainment facilities. Additionally, identification of these viruses in a field survey may trigger reporting requirements and the need for additional safety precautions [107]. Though these viruses may be found infrequently outside of outbreak settings, they remain of great concern due to their potential impact on human wellbeing. Studies using related but less-virulent surrogate viruses or partial virus systems such as minigenomes can be performed at lower biosafety levels, expanding the number of laboratories in which these viruses can be studied. Such studies have contributed in many cases to a better understanding of the pathogens themselves [108–110]. However, the applicability of surrogate data is often unclear, particularly in the absence of studies to bridge to the pathogen of interest [111]. Partial virus systems are useful for focusing in detail on the function or effects of particular viral genes or pathways, but do not provide a holistic view of the full process of viral infection in which multiple cellular and viral pathways interact and influence each other. Therefore, work performed with the viruses themselves in appropriate containment facilities remains critical to a full understanding of their biology and to the development of vaccines and therapeutic interventions to combat their spread.

#### Novel viruses

Less than 1% of the estimated 10^8^ unique viral genotypes [112, 113] globally have been previously described. This is a significant challenge for investigating viral ecology in any environment, including the built environment. Culture-based description of novel viruses is challenged by the necessity for a suitable host cell culture system; the majority of bacterial hosts are unculturable in the lab. Shotgun metagenomics and subsequent assembly of uncultured viral genomes have the potential to resolve this challenge. Standards have recently been developed for publication of an uncultured viral genome, including “virus origin, genome quality, genome annotation, taxonomic classification, biogeographic distribution and in silico host prediction” [114]. Identification of viral hosts is particularly challenging; currently, ~ 95% of the > 800,000 available uncultured viral genomes do not have a putative host [115]. Alternative approaches are needed to elucidate predicted viral hosts (e.g., gene sharing networks) [116]. Ultimately, the ability to explore viral diversity will require funding for this type of basic research.

### Next steps

We have identified three steps that are necessary to grow and support the VIBE research area:

1. While fundamental research into the virome associated with the built environment is valuable, demonstrated impact on human health is necessary to motivate and sustain research support in the VIBE field. One approach could be to prioritize research on specific viruses.
2. We need to determine effective ways to support interactions between different groups of researchers, including architects, engineers, epidemiologists, microbiologists, and physicians. The Sloan Foundation’s Microbiology of the Built Environment program has laid the foundation for such interactions, and we need to ensure that they continue. Certain conferences, such as the Gordon Research Conference on Microbiology of the Built Environment, and special interdisciplinary sessions at conferences on microbiology, exposure, environmental engineering, aerosol science, the built environment, and indoor air quality can help sustain these interactions. Funding opportunities targeted at interdisciplinary groups would, of course, ensure continued collaborations.
3. We need to emphasize the importance and potential high impact of the field and attract more funding to it, although there are challenges and risks associated with supporting a fairly new field that has many unknowns.

Ultimate success of the VIBE field will require an integrated, interdisciplinary approach, demonstrated human health benefits, and risk-tolerant funding opportunities.

## Conclusions

Viruses are ubiquitous in the built environment, and they have been understudied compared to bacteria and fungi. The number of studies on viruses in the built environment is growing; however, new funding opportunities are required to sustain discovery. We hope that identifying these key questions and knowledge gaps will engage funding agencies to spur future research on the highly interdisciplinary topic of viruses in the built environment. Ultimately, understanding viruses in the built environment will lead to improved human and building health.

## Data Availability

Not applicable.
